# Association between prehospital FPS and ROSC in adults with OHCA: A retrospective multicenter study using the German Resuscitation Registry and Intubation Registry (FiPS-CPR)

**DOI:** 10.1007/s00101-022-01193-w

**Published:** 2022-08-29

**Authors:** Sarah Montag, Steffen Herdtle, Samuel John, Thomas Lehmann, Wilhelm Behringer, Christian Hohenstein

**Affiliations:** 1 Klinik für Anaesthesiologie, LMU Klinikum der Universität München, Marchioninistr. 15, 81377 Munich, Germany; 2 Akut- und Notfallmedizin, Krankenhaus Agatharied, Norbert-Kerkel-Platz, 83734 Hausham, Germany; 3 Klinik für Anästhesie und Intensivmedizin, ISAR Klinikum, Sonnenstr. 24–26, 80331 Munich, Germany; 4 Institut für Medizinische Statistik, Informatik und Datenwissenschaften, Universitätsklinikum Jena, Bachstr. 18, 07743 Jena, Germany; 5 Universitätsklinik für Notfallmedizin, MedUni Wien, Währinger Gürtel 18–20, 1090 Vienna, Austria; 6 Interdisziplinäres Notfallzentrum, Zentralklinik Bad Berka GmbH, Robert-Koch-Allee 9, 99437 Bad Berka, Germany

**Keywords:** First-pass intubation success, Airway management, Out-of-hospital cardiac arrest, Resuscitation, Return of spontaneous circulation, Erfolgreicher erster Intubationsversuch, Atemwegsmanagement, Außerklinischer Kreislaufstillstand, Reanimation, Wiedereinsetzen des Spontankreislaufes

## Abstract

**Background:**

Advanced airway management (AAM) is part of the standard treatment during advanced cardiac life support (ACLS). Current studies underline the importance of a first-pass intubation success (FPS) during in-hospital ACLS. It was shown that a failed initial intubation attempt in out-of-hospital cardiac arrest (OHCA) patients in the emergency department is an independent risk factor for the decreased effectiveness of ACLS measured by the return of spontaneous circulation (ROSC).

**Objective:**

This study first examines the association between prehospital FPS and ROSC in adults with OHCA and second identifies factors associated with FPS and ROSC. The initial hypothesis was that FPS would increase the probability of ROSC as well as decrease the time to ROSC.

**Material and methods:**

A retrospective multicenter analysis of 180 adult non-traumatic OHCA patients on whom advanced airway management (AAM) was performed between July 2017 and December 2018 in five different German physician-staffed ambulance stations. For information on FPS the Intubation Registry, and for information on ROSC the German Resuscitation Registry were used. In addition to yes/no questions, multiple answers and free text answers are possible in those questionnaires. The main outcome variables were ‘FPS’, ‘ROSC’ and ‘time to ROSC’. Mann-Whitney tests, χ^2^-tests, Fisher’s exact tests and multivariate binary logistic regressions were used for the statistical evaluation. Demographic factors, characteristics of the performer, selected equipment, laryngoscopy type, intubation method, medications, verification of tube position, respiratory evaluation, complications and time to ROSC were examined with respect to the influence on FPS. Concerning ROSC, the following factors were examined: demographic factors, initial heart rhythm, initial breathing, medications, defibrillation and AAM.

**Results:**

An FPS was recorded in 150 patients (83.3%), and ROSC was achieved in 82 patients (45.5%) after an average time of 22.16 min. There was a positive association between FPS and ROSC (*p* = 0.027). In patients with FPS, a trend for shorter time to ROSC was observed (*p* = 0.059; FPS 18 min; no FPS 28 min). The use of capnography (odds ratio, OR = 7.384, 95% confidence interval, CI 1.886–28.917) and complications during AAM (OR = 0.033, 95% CI: 0.007–0.153) were independently associated with FPS. The independent factor associated with ROSC was FPS (OR = 5.281, 95% CI: 1.800–15.494).

**Conclusion:**

In prehospitally resuscitated adult OHCA patients with AAM, FPS is associated with a higher chance of ROSC.

## Abstract (de)

**Hintergrund:**

Das erweiterte Atemwegsmanagement (AAM) ist Teil der Standardbehandlung bei erweiterten Reanimationsmaßnahmen (ACLS). Aktuelle Studien betonen die Relevanz eines erfolgreichen ersten Intubationsversuches (FPS) während ACLS. Bei Patienten mit außerklinischem Herz-Kreislauf-Stillstand (OHCA) erwies sich ein fehlgeschlagener erster Intubationsversuch als unabhängiger Risikofaktor für die verminderte Wirksamkeit des ACLS – gemessen am Wiedereinsetzen des Spontankreislaufs (ROSC).

**Ziel der Arbeit:**

Diese Studie untersucht den präklinischen Zusammenhang zwischen dem FPS und ROSC bei Erwachsenen mit OHCA. Die initiale Hypothese war, dass bei erzieltem FPS die Wahrscheinlichkeit des ROSC steigt und sich die Zeit bis zum ROSC verringert.

**Material und Methoden:**

Eine retrospektive multizentrische Analyse von 180 erwachsenen Patienten mit nichttraumatischem OHCA, bei denen zwischen Juli 2017 und Dezember 2018 in fünf verschiedenen deutschen ärztlich besetzten Rettungswachen ein AAM durchgeführt wurde. Für Informationen zum FPS wurde das Intubationsregister und für Informationen zum ROSC das Deutsche Reanimationsregister verwendet. Neben Ja/Nein-Fragen sind in diesen Fragebögen auch Mehrfachantworten und Freitextantworten möglich. Die wichtigsten Ergebnisvariablen waren „FPS“, „ROSC“ und „Zeit bis zum ROSC“. Für die statistische Auswertung wurden Mann-Whitney-Tests, χ^2^-Tests, Fisher-Exakt-Test und multivariate binäre logistische Regressionen verwendet. Demografische Faktoren, Charakteristika des Ausführenden, gewählte Ausrüstung, Art der Laryngoskopie, Intubationsmethode, Medikamente, Überprüfung der Tubusposition, respiratorische Bewertung, Komplikationen und Zeit bis zum ROSC wurden im Hinblick auf den Einfluss auf das FPS untersucht. In Bezug auf ROSC wurden folgende Faktoren untersucht: demografische Faktoren, anfänglicher Herzrhythmus, anfängliche Atmung, Medikamente, Defibrillation und AAM.

**Ergebnisse:**

Bei 150 Patienten (83,3 %) wurde ein FPS, bei 82 Patienten (45,5 %) ein ROSC beobachtet. Beide Variablen standen korrelierten positiv miteinander (*p* = 0,027). Bei erzieltem FPS verringerte sich die Zeit bis zum ROSC (*p* = 0,059; FPS 18 min; kein FPS 28 min). Der FPS wurde als unabhängige Variable für den ROSC (OR = 5,281; 95 % KI 1,800–15,494) identifiziert.

**Schlussfolgerung:**

Bei präklinisch wiederbelebten OHCA-Patienten mit AAM ist der FPS mit einer höheren ROSC-Wahrscheinlichkeit assoziiert.

## Introduction and background

Cardiac arrest is one of the most common causes of death. Cardiac arrest is treated by cardiopulmonary resuscitation (CPR): in the case of out-of-hospital cardiac arrest (OHCA), the basic life support, consisting of ventilation and chest compression, should be carried out by medical laypersons immediately after the need for CPR has been established. The medical team arriving usually later continues the CPR with measures of advanced cardiac life support (ACLS). Advanced airway management (AAM) is part of the standard treatment during ACLS [1].

If the first attempt at intubation is successful during AAM, it is called first-pass intubation success (FPS). Current studies underline the importance of FPS during in-hospital ACLS regarding further resuscitation measures [2]. A failed initial intubation attempt during OHCA is an independent risk factor for decreased effectiveness of the whole ACLS [3]. This might be explained by reduced hands-on chest time, which is known to reduce chances for return of spontaneous circulation (ROSC) [1].

The primary aim of this study was to investigate the association between prehospital FPS and ROSC and the occurrence of ROSC and time to ROSC in OHCA patients. The secondary aim was to identify factors associated with FPS and ROSC.

## Methods

### Study design and setting

This was a retrospective multicenter registry study in five different physician-staffed ambulance stations in Germany where AAM is provided by physicians trained in emergency medicine. In this setting, all steps of CPR are performed according to the guidelines of the European Resuscitation Council [4]. This study was approved by the ethics committees of the University Hospitals of Jena (No. 2019-1318-Daten) and Tübingen (No. 116/2019BO2).

### Participants

The inclusion criteria for the patients in the study were non-traumatic OHCA patients aged ≥ 18 years, on whom AAM was provided by prehospital emergency physicians in the German physician-staffed ambulance stations Jena, Meiningen, Tübingen, Wittlich and Wolfenbüttel.

### Data collection and processing

Data were collected during an 18-month period from 1 July 2017 until 31 December 2018, utilizing the local intubation registry, the German Resuscitation Registry, and the emergency medical protocols. All registry data were gathered by online questionnaires.

The intubation registry includes information on AAM (performer, equipment, laryngoscopy, intubation method, medications, verification of tube position, respiratory evaluation, complications) and FPS. The German Resuscitation Registry includes information on CPR (initial heart rhythm, initial breathing, medications, defibrillation) and ROSC.

### Result measurements

The main outcome variables were FPS, ROSC and time to ROSC. Because time to ROSC is not included as variable in the registry, it was calculated by taking the time difference between starting point of CPR and time of ROSC.

### Statistical methods

Continuous variables were presented as mean ± standard deviation (SD) or median and interquartile ranges (IQR), categorical variables as number and percent.

To compare nominal variables with more than 2 categories in univariate analysis we used the χ^2^-test, for continuous variables the Mann-Whitney U test and for binary variables the Fisher’s exact test. Only significant variables were included in the multivariate binary logistic regression and odds ratios (OR) with 95% confidence interval (CI) of the model are reported. All statistical analyses were conducted using the IBM SPSS Statistics software (IBM Corp., Armonk, NY, USA; version 25), the significance level was set at 0.05 for each test.

## Results

### Patient characteristics

During the 18-month study period, 213 patients were recorded in the intubation registry, of which 180 patients were included in the study and analyzed, with most of the exclusions caused by incomplete questionnaires. The study population showed the following demographics: average age of 69.7 years (SD 12.9 years), mean age of 70 years (IQR 60–79.75 years), median body weight of 82.5 kg (IQR 75–95kg) and 71.7% male patients.

An FPS was recorded in 150 patients (83.3%), and ROSC was achieved in 82 patients (45.5%).

### Association between FPS and ROSC and time to ROSC

There was a positive association between FPS and ROSC (*p* = 0.027, Table 1). In multivariate analysis, FPS was the only factor independently associated with ROSC, with an OR of 5.281 (*p* = 0.002, 95% CI 1.800–15.494) (Table 2). In patients with FPS, a statistically non-significant trend for shorter time to ROSC was observed: median 18min (IQR 7–27 min) vs. median 28 min (IQR 21–35 min) (*p* = 0.059, Table 3).

**Table 1:** Comparisons between the groups with and without prehospital ROSC

	ROSC *n* *=* *82 (45.5%)*	No ROSC *n* *=* *98 (54.4%)*	*p*	*n*
*Demographic factors*				
Age, years, median (IQR)	71.5 (57–78.5)	68.3 (50–80.25)	0.148	180
Sex, male, no. (%)	55 (67.1)	74 (75.5)	0.246	129
Body weight, kg, median (IQR)	86.6 (73.7–90)	89.5 (75–100)	0.057	180
*Initial heart rhythm, n* *=* *180*				
Asystole, no. (%)	38 (46.3)	72 (73.5)	< 0.001	110
PEA, no. (%)	13 (15.9)	11 (11.2)	0.386	45
Ventricular fibrillation, no. (%)	31 (37.8)	14 (14.3)	< 0.001	24
Pacemaker, no. (%)	0 (0)	1 (1)	1.000	1
*Initial breathing, n* *=* *180*				
Apnea, no. (%)	60 (73.2)	91 (92.9)	< 0.001	151
Gasping, no. (%)	19 (23.2)	5 (5.1)	< 0.001	24
Ventilation, no. (%)	3 (3.6)	2 (2)	0.661	5
*Medications, n* *=* *180*				
Amiodarone, no. (%)	55 (67.1)	80 (81.6)	0.037	135
Defibrillation^a^	–	–	0.227	76
*AAM, n* *=* *180*				
FPS, no. (%)	74 (90.2)	76 (77.6)	0.027	150
No FPS, no. (%)	8 (9.8)	22 (22.4)		30

*ROSC* return of spontaneous circulation, *IQR* interquartile range, *PEA* pulseless electrical activity, *AAM* advanced airway management, *FPS* first-pass intubation success

^a^ Variables: 0, 1, 2–3, 4–6, 7–9, > 9 defibrillation attempts

**Table 2:** Multivariate binary logistic regressions for FPS and ROSC

	Variable	*p*	OR	CI
FPS	Body weight, per kg	0.278	0.988	0.967–1.010
	Throat suction	0.400	0.551	0.137–2.211
	Capnography	0.004	7.384	1.886–28.917
	Grade I CL	0.152	5.628	0.528–59.960
	Grade III CL	0.064	0.210	0.040–1.097
	Grade IV CL	0.287	0.295	0.031–2.797
	Complications	< 0.001	0.033	0.007–0.153
ROSC	Asystole	0.343	0.632	0.244–1.632
	Ventricular fibrillation	0.125	2.661	0.762–9.294
	Apnea	0.478	0.503	0.075–3.364
	Gasping	0.413	2.436	0.289–20.543
	Amiodarone	0.953	1.028	0.414–2.554
	FPS	0.002	5.281	1.800–15.494

*OR* odds ratio, *CI* 95% confidence interval, *FPS* first-pass intubation success, *CL* Cormack and Lehane, *ROSC* return of spontaneous circulation

**Table 3:** Comparisons between the groups with and without FPS

	FPS *n* *=* *150 (83.3%)*	No FPS *n* *=* *30 (16.7%)*	*p*	*n*
*Demographic factors*				
Age, years, median (IQR)	71 (60.75–80)	65.6 (58.5–78.25)	0.186	180
Sex, male, no. (%)	104 (69.3)	25 (83.3)	0.182	129
Body weight, kg, median (IQR)	80 (75–90.5)	90 (80–106.25)	0.044	180
*Performer*				
Level of education^a^	–	–	0.180	165
Specialization^b^	–	–	0.335	149
*Selected equipment, n* *=* *177*				
ET, no. (%)	124 (83.8)	27 (93.2)	0.197	151
LT, no. (%)	23 (15.5)	1 (3.4)		24
LM, no. (%)	1 (0.7)	1 (3.4)		2
*Laryngoscopy, n* *=* *107*				
VL, no. (%)	28 (31.5)	7 (38.9)	0.062	35
VL and DL, no. (%)	0 (0)	1 (5.5)		1
DL, no. (%)	61 (68.5)	10 (55.6)		71
*Method, n* *=* *179*				
Oral, no. (%)	19 (12.8)	4 (13.3)	0.901	23
Oral RSI, no. (%)	129 (86.6)	26 (86.7)		155
Oral DSI, no. (%)	1 (0.6)	0 (0)		1
*Throat suction, n* *=* *180*				
Throat suction, no. (%)	40 (26.7)	18 (60)	0.001	58
*Medications, n* *=* *180*				
Analgesics^c^, no. (%)	5 (100)	11 (100)	0.130	16
Sedatives^d^, no. (%)	6 (100)	11 (100)	0.692	17
Muscle relaxants^e^, no. (%)	4 (100)	12 (100)	0.585	16
*Verification of tube position, n* *=* *171*				
Auscultation, no. (%)	127 (32)	17 (40.5)	0.351	144
Capnography, no. (%)	122 (30.7)	8 (19)	< 0.001	130
Capnometry, no. (%)	51 (12.8)	7 (16.7)	1.000	58
Fiber optic/bronchoscopy, no. (%)	1 (0.3)	0 (0)	1.000	1
Intubation under visibility, no. (%)	96 (24.2)	10 (23.8)	0.102	106
*Respiratory evaluation, CL, n* *=* *117*				
Grade I, no. (%)	42 (44.7)	2 (8.7)	0.011	44
Grade II, no. (%)	42 (44.7)	9 (39.1)	0.827	51
Grade III, no. (%)	7 (7.4)	6 (26.1)	0.010	13
Grade IV, no. (%)	3 (3.2)	6 (26.1)	0.010	9
*Complications*^*f*^*, n* *=* *180*				
Complication, no. (%)	7 (4.7)	19 (63.3)	< 0.001	26
*ROSC, n* *=* *82*				
Time to ROSC, median (IQR)	18 (7–26.75)	28 (21.25–35)	0.059	77

*FPS* first-pass intubation success, *IQR* interquartile range, *CL* Cormack and Lehane, *ROSC* return of spontaneous circulation, *AAM* advanced airway management

^a^ Variables: junior doctor, medical specialist, senior doctor, head doctor, paramedic

^b^ Variables: general medicine, anesthesiology, internal medicine, intensive care, emergency medicine, trauma surgery, other surgery

^c^ Variables: fentanyl, morphine, ketamine/esketamine, fentanyl and ketamine/esketamine

^d^ Variables: midazolam, propofol, midazolam and etomidate

^e^ Variables: succinylcholine, rocuronium

^f^ Variables: failed intubation, aspiration, hypoxia, hypotension, displacement, laryngospasm, insufficient AAM

### Factors associated with FPS

Patients were divided into two groups based on the presence of prehospital FPS (FPS, *n* = 150, 83.3%; No FPS, *n* = 30, 16.7%, Table 3).

In univariate analysis (Table 1), the following factors were associated with FPS: body weight (*p* = 0.044), throat suction (*p* = 0.001), verification of tube position (*p* < 0.001), airway assessment according to Cormack and Lehane (*p* < 0.001) and complications during intubation (*p* < 0.001). The following factors were not associated with FPS: age (*p* = 0.186), sex (*p* = 0.182), level of education of performer (*p* = 0.180), specialization of performer (*p* = 0.335), selected intubation equipment (*p* = 0.197), type of laryngoscopy (*p* = 0.062), intubation method (*p* = 0.901), specific medications (analgesics, *p* = 0.130; sedative, *p* = 0.692; muscle relaxants, *p* = 0.585) and time to ROSC (*p* = 0.059).

In multivariate analysis (Table 2), the use of capnography (OR = 7.384, 95% CI: 1.886–28.917) and complications during AAM (OR = 0.033, 95% CI: 0.007–0.153) were independently associated with FPS.

### Factors associated with ROSC

Patients were divided into two groups based on the presence of prehospital ROSC (ROSC, *n* = 82, 45.6%; No ROSC, *n* = 98, 54.4%; Table 1). The average time to ROSC was 22.16 min (SD 12.7 min), the mean time was 20 min (IQR 14.5–28.5 min).

In univariate analysis (Table 1), the following factors were associated with ROSC: initial hearth rhythm (*p* = 0.001), initial breathing (*p* = 0.001), amiodarone (*p* = 0.037) and FPS (*p* = 0.027). The following factors were not associated with ROSC: age (*p* = 0.148), sex (*p* = 0.246), body weight (*p* = 0.057) and number of defibrillations (*p* = 0.227).

In multivariate analysis (Table 2), only FPS was independently associated with ROSC (OR = 5.281, 95% CI: 1.800–15.494).

## Discussion

This study showed that in adult OHCA patients with AAM prehospital FPS was associated with a higher chance of ROSC. Factors associated with FPS positively were the use of capnography for confirmation of tube position, and no complications during AAM. Only FPS was a factor associated with ROSC.

In recent studies, FPS has been in the spotlight, e.g. a systematic review examined the emergency AAM in general and concluded that preclinical physicians achieved an FPS rate of 71.2–87.5% [2]. The FPS rate in this study (83.3%) during prehospital CPR is in line with these results. Since there was no connection between the FPS and the level of education of performer and specialization of performer in this study, one can assume good intubation expertise on the part of the emergency physicians for the data examined. Compared to pan-European data (32.7%) [9] the rate of prehospital ROSC (45.6%) was higher. This might be explained by the fact that in this study AAM was mostly provided by emergency physicians and not by paramedics.

Regarding FPS, the negative association of body weight to FPS can be explained by AAM itself becoming more difficult [10]. Using capnography for confirmation of correct tube position is required in the current resuscitation guidelines as the treatment standard [1]. The results of this study support this fact, because using capnography was independently associated with higher chance of FPS. The available data also show that the application rate of capnography was not 100%. This is probably due to the fact that the questionnaire differentiates between capnography and capnometry and is marginally misleading when it is filled out. This fuzziness should be examined more profoundly in the future.

Over the past few years, many studies have been carried out researching the best equipment for AAM [5–8]. This study cannot draw any sound conclusions about the best equipment, because ETI is overpresented (85.3% of all intubations).

Regarding complications during AAM (complication rate 14.2%), the most common documented was failed intubation followed by aspiration. Statistically, in this study complications during AAM were independently associated with FPS. Here, future studies should clarify the connection, because it is not clear whether the FPS failed due to complications during the AAM or whether an FPS was present before the emergency physicians noticed those complications (e.g. later aspiration). Further improvement of the questionnaires to depict the chronological sequence of event should occur.

The findings of this study show that a shockable rhythm and present gasping have a positive effect on probability of ROSC by intervening in the circulatory arrest in an earlier more favorable phase, which is consistent with other studies [3, 11, 12]. Compared to other studies, time to ROSC is slightly longer, which can be justified by methodological differences on calculating time to ROSC [11, 13]. Therefore, it would be desirable to integrate the variable time to ROSC into the resuscitation registers in order to ensure clear comparability.

The results confirm the significant positive association between prehospital FPS and ROSC [3, 14], with the distinctiveness that this study found this association in a setting with physician-staffed ambulance stations. If FPS was achieved, time to ROSC was lowered by an average of 10 min [3].

As prolonged intubation attempts usually occur while stopping chest compressions, an extended no-flow time might reduce the chance to achieve a ROSC and therefore affirm the importance of a fast FPS. Other reasons for the lower rate of ROSC might be a delay of other necessary interventions such as defibrillation or detecting and treating reversible causes of the cardiac arrest.

In future this study design should be repeated with larger power to see whether there is a statistically significant difference in time to ROSC depending on prehospital FPS.

Additionally, the length of the interruptions for intubating (if present) while performing chest compressions and subsequent disturbances if the ACLS algorithm should be further queried in the questionnaires. To this end, the questionnaires could be modified so that the medical staff could assess the effectiveness of their chest compressions and the amount of time they spent on resuscitation on a scale.

### Limitations

This study has several limitations. First, the results are based on a retrospective analysis of 2 registries with the possible bias by the intrinsic design itself.

Second, the full treatment time was not always documented (start and end), therefore valid statements about survival times could not be made.

Third, this study only looked at appearance of preclinical ROSC. It is not possible to draw any conclusions as to whether the patients achieved a ROSC later in the hospital and no evaluation of neurological outcome.

Fourth, the informative value is limited in that the group sizes “FPS” and “No FPS” differ greatly from one another in quantitative terms. Attention should be paid to greater power in future investigations and possibly more locations to be included.

## Conclusion

These results indicate that in adult OHCA patients with AAM, prehospital FPS is associated with a higher chance of ROSC. Additionally, the use of capnography and no complications during AAM were positive associated with the successful first-pass attempt.

## Declarations

This retrospective study was performed after consultation with the ethics committee of the university clinics in Jena and Tübingen and in accordance with national legal requirements.
